# Evaluating the psychometric quality of school connectedness measures: A systematic review

**DOI:** 10.1371/journal.pone.0203373

**Published:** 2018-09-11

**Authors:** Amy Hodges, Reinie Cordier, Annette Joosten, Helen Bourke-Taylor, Renée Speyer

**Affiliations:** 1 School of Occupational Therapy, Social Work and Speech Pathology, Curtin University, Perth, Western Australia, Australia; 2 School of Allied Health, Australian Catholic University, Melbourne, Victoria, Australia; 3 School of Primary and Allied Health, Medicine, Nursing and Health Sciences, Monash University, Frankston, Victoria, Australia; 4 Department Special Needs Education, University of Oslo, Oslo, Norway; 5 Department of Otorhinolaryngology and Head and Neck Surgery, Leiden University Medical Centre, Leiden, the Netherlands; Kyoto University, JAPAN

## Abstract

**Introduction:**

There is a need to comprehensively examine and evaluate the quality of the psychometric properties of school connectedness measures to inform school based assessment and intervention planning.

**Objective:**

To systematically review the literature on the psychometric properties of self-report measures of school connectedness for students aged six to 14 years.

**Methods:**

A systematic search of five electronic databases and gray literature was conducted. The COnsensus-based Standards for the selection of heath Measurement INstruments (COSMIN) taxonomy of measurement properties was used to evaluate the quality of studies and a pre-set psychometric criterion was used to evaluate the overall quality of psychometric properties.

**Results:**

The measures with the strongest psychometric properties was the School Climate Measure and the 35-item version Student Engagement Instrument exploring eight and 12 (of 15) school connectedness components respectively.

**Conclusions:**

The overall quality of psychometric properties was limited suggesting school connectedness measures available require further development and evaluation.

## Introduction

The concept of school connectedness has received growing attention from researchers and educators in recent years due to its reported impact on health, social and academic outcomes [1–3]. Students who have a stronger sense of school connectedness are more likely to: engage in socially appropriate behaviours; have higher levels of self-esteem; obtain better grades; display acceptable conduct at school; and are more likely to graduate than students with a lower sense of school connectedness [4–7]. Longitudinal research suggests that students’ sense of school connectedness in early schooling increases engagement in risk behaviour’s such as smoking, marijuana use, alcohol consumption and sexualised behaviour in later schooling [2, 8–10]. Recent evidence also suggests that students with a lower sense of school connectedness are more likely to experience clinical anxiety and depression during their schooling and in later life [3, 11].

School connectedness presents an attractive focus for educators, school psychologists and researchers as it is a subjective concept that is amenable to change through the provision of appropriate school based supports [8, 12]. School connectedness literature is being used widely to inform the development of school based interventions, as well as inform educational policy and reform [13, 14]. The Australian Early Years Learning Framework [15] is an example of this; centred around the notion that for students to experience learning that is engaging and supportive of success in later life, they need to first have a sense of belonging to their school community. As such, there is a need for valid and reliable measures to assess the effectiveness of school based interventions targeting school connectedness, in order to minimise the long term documented impacts of reduced school connectedness on students’ academic success and socio-emotional wellbeing. Furthermore, access to school connectedness measures with sound psychometric properties will assist in gaining further evidence to support the use of school based interventions and assist in informing educational policy and reform.

### School connectedness: Theoretical underpinnings and definition

Despite growing interest in the concept of school connectedness, there is considerable debate regarding the definition of school connectedness. Many terms have been used inter-changeably in the literature to describe school connectedness including school climate, belonging, bonding, membership and orientation to school [16, 17]. As a result, the operationalisation and measurement of school connectedness has been challenging.

Theoretical models of school connectedness are most commonly embedded within psychology literature. Deci and Ryan’s [18] self-determination theory is regularly referred to within school connectedness literature [19–23]. This theory proposes that for an individual to be motivated and to function optimally, a set of psychological needs such as relatedness, competence and autonomy must be supported [18]. Relatedness refers to a need to feel a sense of belonging with peers and teachers [18, 24]. Competence is the need to feel capable of learning and autonomy is the need to feel that you have choice and control at school [18, 24]. These three innate psychological traits are often cited to account for human tendencies to “…engage in activities, to exercise capacities and to pursue connectedness in social groups” [24]; all of which are foundational skills in developing students’ sense of school connectedness. Self-determination theory suggests that students with a strong sense of relatedness or belonging to their peers, teacher and school community are in a better position to learn and more likely to perform better at school due to improved wellbeing and resilience. Furthermore, students who perceive their school environment to be fair, ordered and disciplined and who feel in control of their academic outcomes at school, are more likely to engage and feel connected at school. Deci and Ryan’s [18] self-determination theory illuminates the impact affective, behavioural and cognitive factors have in supporting or hindering a student’s sense of school connectedness.

Early research relating to school connectedness has focused on affective aspects of school connectedness [17, 25]. Affective engagement, also referred to as psychological and emotional engagement, refers to a student’s feelings towards his/her school, learning, teachers and peers [17, 25, 26]. Affective engagement is accurately captured in Goodenow’s [27] definition of school connectedness, which is the “…extent to which a student feels personally accepted, respected, included and supported by others” [27] in the school environment. This definition, however, does not take into consideration behavioural and cognitive factors that can also impact a student’s sense of school connectedness, which have been explored in more recent school connectedness literature. Behavioural engagement includes observable student actions of participation while at school and is investigated through student conduct, effort and participation [5, 28, 29]. Conversely, cognitive engagement includes students’ perceptions and beliefs associated with school and learning [5, 28, 29]. That is, to feel connected to school the student must be actively involved in classroom and school activities, including school organised extra-curricular activities, and actively think about how they can involve themselves in the learning process at school. Wingspread’s Declaration of School Connections [30], which describes school connectedness as a “…belief by students that adults in the school community care about students learning and about them as individuals and can be represented by high academic expectations from teachers with support for learning, positive teacher-student interactions and feelings of safety” [30], more accurately captures behavioural and cognitive aspects of school connectedness.

Several reviews have focused on defining the meta-construct of school connectedness [7, 25, 31]. These reviews highlight that the construct of school connectedness has evolved over time—from a relatively simple construct focusing on students’ general feelings towards school; to a more complex multi-dimensional construct comprising not only students’ feelings towards school, but also their perceptions and beliefs towards school and learning, and their involvement in classroom and playground activities and school events. Researchers in the field postulate that definitions of school connectedness should include the triad of indicators (i.e., affective, behavioural, and cognitive) and facilitators (i.e., personal and contextual factors) that influence connectedness [25]. Indicators “…convey a student’s degree or level of connection with learning while facilitators are factors that influence the strength of the connection” [25]. Although this definition has been proposed, authors of this study have not found a definition of school connectedness that fully encapsulates all of these components. Following an extensive review of the literature, authors of the study thematically categorised factors contributing towards students’ sense of school connectedness under affective, cognitive and behavioural domains illustrated in Table 1. For the purposes of this review, these domains and concepts will be subsumed under the broader construct of school connectedness. Collectively, the concepts in Table 1 are critical dimensions of students’ experiences in school. Together, they are essential in promoting student development and overall academic success. These concepts are often targeted within individual and school wide interventions strategies. As such, there is a need for measures that assess these school connectedness domains and constructs both cross-sectionally and longitudinally.

**Table 1:** School connectedness domains and constructs.

Affective	Cognitive	Behavioural
Feelings of acceptance, inclusion and belonging Feelings of respect and being respected Valuing the importance of school Sense of safety Sense of autonomy and independence Feeling competent in academic abilities.	Perceptions of the quality of teacher relationships and support Perceptions of the quality of peer relationships and support Perceptions of the quality of academic support Perceptions of discipline, fairness, order in the school Perceptions of the value parents place on school and support engagement	Actual involvement, participation or engagement (including classroom and playground activities, school organised extra-curricular activities or school events) Level of effort or persistence Positive or negative conduct Degree of interest or motivation towards school

### Measuring school connectedness

Not surprisingly, given the difficulties in defining school connectedness, there are various ways in which this concept has been measured. The differences in the way the concept is measured are theoretical and methodological. The theoretical background of the researcher often determines how school connectedness is measured. For example, Jimerson, Campos and Grieif [31] identify and assess student motivation as an affective indicator of school connectedness with a background in psychology; while Fredricks, Blumenfeld and Paris [7] identify it as a cognitive indicator with a background in educational psychology. While motivation is an intrinsic process, it manifests itself extrinsically through student behaviour [32]. Therefore, authors of this study have categorised student interest or motivation as a behavioural indicator of school connectedness (see Table 1).

The purpose of assessing school connectedness often determines how the construct is measured. Some measures have been developed specifically for the school context (e.g., What’s Happening In This School [33]), whereas others extend their exploration to the home and community environment with subscales or items that refer to school (e.g., Adolescents Sense of Wellbeing Related to Stress [34]). Some measures have been developed specifically to assess students’ sense of school connectedness in particular subjects such as maths, science or physical education (e.g., What’s Happening In This Class (Singapore version) [35]). Some measures focus on assessing an individual student’s sense of connectedness (e.g., Student Engagement Instrument [36]), whereas others aim to assess an individual’s perception of connectedness at a classroom or school level (e.g., Classroom Environment Scale [37], Classroom Peer Context Questionnaire [38]). Schools conducting research into school connectedness will often tailor their measurement approach based on their needs; for example, whether they want to gain an understanding of their schools sense of connectedness to inform funding allocation, versus whether they want to identify individual at-risk students to inform the provision of school supports [39].

There is debate within the literature regarding whether self-report or proxy report measures should be used when evaluating school connectedness [40]. Many would argue the subjective nature of school connectedness makes it less amenable to third party report [17, 31]. For example, the teacher may observe the student to play with peers or engage in the curriculum, but the student themselves, for whatever reason, may not feel like they are a part of their school community. Self-report measures help to depict the student’s personal perception of their experience at school. Teacher-report methods may be more suitable in capturing behavioural components of school connectedness such as the students’ level of effort or persistence at school that can be objectively observed [41]. As previously mentioned, students will experience a sense of connectedness when their needs of autonomy, competence and relatedness are met within the school environment [24]. The assumption is that students’ feelings of being included and accepted at school, as well as the perception they are making important contributions to the school community, help to create and maintain feelings of connectedness. Therefore, in order to gain an accurate depiction of students’ sense of school connectedness, the use of student self-report measures is warranted and will be the focus of this particular review.

The differences in the way school connectedness is defined makes it difficult to compare measures to each other in an attempt to identify the most valid and reliable tool to use in the school context. As children spend more time in schools than any other place outside their homes, it is important to be able to validly and reliably assess student experiences within school so that appropriate supports can be provided [39]. Furthermore, it is important to be able to reliably measure this construct with students in early primary school, to prevent or minimise the long term documented impacts of reduced school connectedness on student outcomes.

The COSMIN taxonomy has been successfully applied to more than 560 systematic reviews [42, 43]. The COSMIN checklist is a standardised tool that can be used to critically appraise the methodological quality of studies reporting on the psychometric properties of measures [43]. The COSMIN checklist was chosen for this systematic review as it has been developed following extensive international consultation and consensus among experts in the field of psychometrics and clinimetrics. The COSMIN was used in the current review to compare the psychometric properties of existing school connectedness measures, originally developed in English that capture affective, cognitive and behavioural domains of school connectedness using self-report methods for students aged six to 14 years of age. It is expected that this systematic review will assist in the choice of instruments measuring school connectedness, by providing an objective account of the strengths and weaknesses of self-report measures available for school aged children.

## Methods

The Preferred Reporting Items for Systematic Reviews and Meta-Analyses (PRISMA) statement guided the methodology and writing of this systematic review. The PRISMA statement is a 27–item checklist that is deemed essential in the transparent reporting of systematic reviews [44]. A completed PRISMA checklist for the current review is accessible (see S1 Table).

### Eligibility criteria

Research articles, published manuals and reports detailing the psychometric properties of self-report instruments designed to measure school connectedness of students aged six to 14 years of age were deemed eligible for inclusion in this review. To be included, abstracts and instruments needed to address all three school connectedness domains (i.e., behavioural; affective and cognitive); address at least five of 15 concepts within school connectedness domains (see Table 1); provide validity evidence for students aged six to 14 years of age; be specific to the school context; have psychometrics properties published within the last 20 years; and be written in English. Psychometrics properties published more than 20 years ago were deemed out-dated. Measures were excluded if the full text of the article was not retrievable; they were specific to a subject area (e.g., maths or science) or a student population (e.g., students with craniofacial abnormalities). Measures that provided validity evidence for students requiring special education assistance were included in the review, as long as the sample also included typically developing students. Dissertations, conference and review papers were excluded as they are not peer reviewed, and the search yielded sufficient results.

### Information sources

The first systematic literature search was performed on the 13^th^ June 2016 by two authors using the following five electronic databases: CINAHL, Embase, ERIC, Medline, PsycINFO. Subject headings and free text were used when searching each database. A gray literature search was also conducted using Google Scholar and PsycEXTRA between the 21^st^ and 27^th^ July 2016 to identify additional measures. See S2 Table for a complete list of search terms used across all searches. A second literature search was conducted on the 18^th^ September 2016 using the title of the measure and its acronym in CINAHL, Embase, ERIC, Medline and PsycINFO to identify additional psychometric articles not identified in the first search. To be comprehensive, websites of publishers of assessments in education and social science such as Pearson Education, ACER and Academic Therapy Publications were searched.

### Study selection

**Fig 1:**
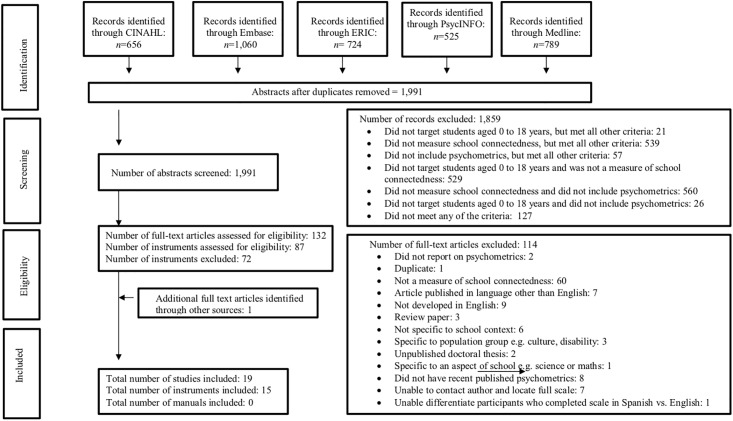
Flow diagram of the reviewing process according to PRISMA [44].

Abstracts were reviewed using three dichotomous scales to determine (a) if the study involved students aged between 0 and 18 years (yes/no), (b) if the instrument measured school connectedness or related terms (e.g., group membership, learner engagement, school community relationship, student participation, school involvement) (yes/no) and (c) if the study reported on the psychometric properties of the measure (yes/no). Results from the three dichotomous scales were then combined to generate a single ordinal scale from 0 to 3; 0 indicating the abstract did not meet any criteria and 3 indicating the abstract met all three criteria. A random sample of 40% of abstracts was generated using an electronic random allocator (www.random.org). Based on previous systematic reviews using COSMIN [45–47], this percentage was deemed sufficient to detect systematic error. The random sample was reviewed by the primary author and an independent rater to establish inter-rater reliability. Inter-rater reliability between raters was deemed excellent: Weighted Kappa = 0.814 (95% CI: 0.791–0.836). Abstracts that did not meet any of the criteria or met only one of the criteria were excluded from the study. Abstracts that met two or three of the criteria were reviewed a second time and discussed by the primary author and independent rater to gain consensus and ensure only studies meeting all eligibility criteria were included in full text review. The primary author then rated the remaining abstracts and 132 full texts articles meeting all three criteria. Articles were excluded if the full text did not meet criteria (see Fig 1). Scoring a random sample of abstracts first, allowed the researcher to learn from the process and avoid systematic errors.

### Data collection process and data extraction

Information from articles were extracted under the following descriptive categories: purpose of the measure, number of subscales, total number of items, response options and time to complete, article reference and sample characteristics. The information extracted from articles was guided by the Cochrane Handbook for Systematic Reviews [48] Section 7.3a and the Systematic Reviews Centre for Reviews and Dissemination [49].

### Methodological quality

The methodological quality of included studies was assessed using the COSMIN taxonomy of measurement properties and definitions for health-related patient reported outcomes [43, 50]. The COSMIN checklist is a standardised tool and consists of nine domains: internal consistency, reliability (including test-retest reliability, inter-rater reliability and intra-rater reliability), measurement error, content validity (including face validity), structural validity, hypotheses testing, cross cultural validity, criterion validity and responsiveness [43]. Refer to Table 2 for the definitions of all psychometric properties as defined by the COSMIN statement [50]. Responsiveness was not evaluated as a psychometric property as it would have increased the size of the review exponentially and was deemed outside the scope of this review. Criterion validity was also not evaluated due to the absence of a ‘gold standard’ measure of school connectedness. Cross-cultural validity was not evaluated as instruments included in the review were developed and published in English. Interpretability is not considered to be a psychometric property under the COSMIN framework and was therefore not described or evaluated in this review.

**Table 2:** COSMIN definitions of domains, psychometric properties and aspects of psychometric properties for health-related patient-reported outcomes adapted from Mokkink et al. [50].

Psychometric property	Definition^a^
**Validity:** the extent to which an instrument measures the construct/s it claims to measure.	
**Content validity**	The degree that the content of an instrument adequately reflects the construct to be measured.
Face validity^b^	The degree to which instrument (items) appear to be an adequate reflection of the construct to be measured.
**Construct validity**	The extent to which the scores of an instrument are consistent with hypotheses, based on the assumption that the instrument is a valid measure of the construct being measured.
Structural validity^c^	The extent to which instrument scores adequately reflect the dimensionality of the construct to be measured.
Hypothesis testing^c^	Item construct validity.
Cross cultural validity^c^	The degree to which the performance of items on a translated or culturally adapted instrument are an adequate reflection of the performance of the items in the original version of the instrument.
**Criterion validity**	The degree to which the scores of an instrument satisfactorily reflect a “gold standard”.
**Responsiveness**	The capability of an HR-PRO instrument to detect change in the construct to be measured over time.
**Interpretability**^d^	The extent to which qualitative meaning can be given to an instrument’s quantitative scores or score change.
**Internal consistency**	The level of correlation amongst items.
**Reliability**	The proportion of total variance in the measurements due to “true” differences amongst patients.
**Measurement error**	The error of a patient’s score, systematic and random, not attributed to true changes in the construct measured.

Notes.

^a^Applies to Health-Related Patient-Reported Outcomes (HR-PRO) instruments.

^b^Aspect of content validity under the domain of validity.

^c^Aspects of construct validity under the domain of validity.

^d^Interpretability is not considered a psychometric property.

Each domain of the COSMIN checklist includes 5 to 18 items focusing on various aspects of study design and statistical analyses. A 4–point rating scale proposed by Terwee et al. [51] enables an overall methodological quality score from poor to excellent, to be obtained for each measure. Terwee et al. [51] suggests taking the lowest rating of any item in the domain as the final quality rating, however this makes it difficult to differentiate between subtle psychometric qualities of assessments. Therefore a revised scoring system was applied and presented as a percentage: Poor (0–25%), Fair (25.1%–50.0%), Good (50.1%–75%) and Excellent (75.1–100%) [47]. As some COSMIN items only have an option to rate as good or excellent, the total score for each psychometric property was calculated using the formula detailed below, to accurately capture the quality of psychometric properties [43]:

$$Total\;score\;per\;psychometric\;property\;=\;\frac{\left(Total\;score\;obtained-Min\;score\;possible\right)}{\left(Max\;score\;possible-Min\;score\;possible\right)}\times 100\%$$

After the studies were assessed for methodological quality, the quality of psychometric properties were evaluated using modified criteria by Terwee [51] and Schellingerhout et al. [52]. A summary of the criteria used for rating the quality of internal consistency, content validity, structural validity and hypothesis testing is detailed in Table 3. Finally, each measurement property for all instruments was given an overall score using criteria set out by Schellingerhout [52]. An overall quality rating was created by combining the study quality scores measured by COSMIN and the psychometric quality ratings as measured by Terwee et al. (2007) and Schellingerhout [52]. This method has been used successfully in previous psychometric reviews [45, 53]. The COSMIN checklist [51] and Terwee [51] and Schellingerhout et al. [52] criteria accommodates studies that use both Classical Test Theory (CTT) and Item Response Theory (IRT) methodology.

**Table 3:** Criteria of psychometric quality rating based on Terwee et al. [50] and Schellingerhout et al. (2012).

Psychometric property	Score^a^	Quality criteria^b^
**Content validity**	+	A clear description is provided of the measurement aim, the target population, the concepts that are being measured, and the item selection and target population and (investigators or experts) were involved in item selection
	?	A clear description of above-mentioned aspects is lacking or only target population involved or doubtful design or method
	-	No target population involvement
	±	Conflicting results
	NR	No information found on target population involvement
	NE	Not evaluated
**Structural validity**^c^	+	Factors should explain at least 50% of the variance
	?	Explained variance not mentioned
	-	Factors explain <50% of the variance
	±	Conflicting results
	NR	No information found on structural validity
	NE	Not evaluated
**Hypothesis testing**^c^	+	Specific hypotheses were formulated AND at least 75% of the results are in accordance with these hypotheses
	?	Doubtful design or method (e.g., no hypotheses)
	-	Less than 75% of hypotheses were confirmed, despite adequate design and methods
	±	Conflicting results between studies within the same manual
	NR	No information found on hypotheses testing
	NE	Not evaluated
**Internal consistency**	+	Factor analyses performed on adequate sample size (7 * # items consistency and ≥100) AND Cronbach’s alpha(s) calculated per dimension and Cronbach’s alpha(s) between 0.70 and 0.95
	?	No factor analysis OR doubtful design or method
	-	Cronbach’s alpha(s) <0.70 or >0.95, despite adequate design and method
	±	Conflicting results
	NR	No information found on internal consistency
	NE	Not evaluated
**Reliability**	+	ICC or weighted Kappa ≥0.70
	?	Doubtful design or method (e.g., time interval not mentioned)
	-	ICC or weighted Kappa < 0.70, despite adequate design and method
	±	Conflicting results
	NR	No information found on reliability
	NE	Not evaluated
**Measurement error**^d^	+	MIC < SDC OR MIC outside the LOA OR convincing arguments that agreement is acceptable
	?	Doubtful design or method OR (MIC not defined AND no convincing arguments that agreement is acceptable)
	-	MIC ≥ SDC OR MIC equals or inside LOA, despite adequate design and method;
	±	Conflicting results
	NR	No information found on measurement error
	NE	Not evaluated

Notes.

^a^Scores: + = positive rating, ? = indeterminate rating, - = negative rating, ± = conflicting data, NR = not reported, NE = not evaluated (for study of poor methodological quality according to COSMIN rating, data are excluded from further evaluation).

^b^Doubtful design or method is assigned when a clear description of the design or methods of the study is lacking, sample size smaller than 50 subjects (should be at least 50 in every subgroup analysis), or any important methodological weakness in the design or execution of the study.

^c^Hypothesis testing: all correlations should be statistically significant (if not, these hypotheses are not confirmed) AND these correlations should be at least moderate (r > 0.5).

^d^Measurement error: MIC = minimal important change, SDC = smallest detectable change, LOA = limits of agreement.

To maximise consistency of ratings, the fifth author of this study who has extensive experience in the area provided training to the primary author and an independent rater on how to complete the COSMIN checklist and to determine the quality of the psychometric properties. The first author scored all the papers. A random selection of 40% of COSMIN ratings and all psychometric quality ratings were scored by an independent rater. Both raters met until 100% consensus was achieved when ratings differed in category. The fifth author met with the two raters to resolve differences in ratings when a consensus could not be reached (Weighted Kappa: 0.886, 95% CI: 0.823–0.948).

### Data items, risk of bias and synthesis of results

All data items for each measure were obtained. Items that were not reported were recorded as ‘NR’. Risk of bias was assessed at an individual study level using the COSMIN checklist. Studies that obtained a high rating were deemed to be at low risk of bias and studies that obtained a low rating were deemed at high risk of bias. Psychometric properties only received a ‘positive’ or ‘negative’ rating if clear and appropriate methodology was reported. If unclear or inappropriate methodology was used, an ‘indeterminate’ rating was recorded; providing further evidence for risk of bias. Ratings from individual studies and psychometric properties were then combined to create an overall rating for each psychometric property of each measure. Risk of bias is subsumed into final results.

## Results

### Systematic literature search

A total of 3,754 abstracts were retrieved from database searches, including duplicates. The total abstracts from subject heading and free text word searches across databases were: CINAHL = 656, Embase = 1,060, ERIC = 724, Medline = 789, PsycINFO = 525. Reference lists of included articles were searched for additional literature. A total of 1,763 duplicates were identified across the five databases and removed. After the removal of duplicate abstracts, a total of 1,991 articles were screened for inclusion in the review. Of these studies, 132 full text articles on 87 measures were assessed for eligibility. Of these 87 measures, 15 met the inclusion criteria and 72 were excluded. Refer to S3 Table for an overview of the 72 excluded instruments and the reasons for exclusion. The references of two manuals were identified for two included instruments; however, because they were irretrievable they were not included in the review. Therefore, psychometric properties of 15 measures were obtained, which were assessed using 18 research articles and 1 research report. Fig 1 illustrates the reviewing process according to PRISMA.

### Included school connectedness measures

Table 4 summarises characteristics of 15 measures that met inclusion criteria and articles reporting on psychometric properties. All measures were developed and validated with typically developing students from a range of ethnic and socio-economic backgrounds in the United States, except for one, which was developed in New Zealand [54]. The majority of measures were developed with an adolescent sample (12 to 18 years), with only a small number of measures developed and validated with students under the age of 12 years [55, 56]. Only three measures extended their samples to include students receiving special education services; however, these students made up less than 15% of the total sample [55, 57–59]. The majority of studies had large sample sizes, with the median sample size being 1,642 (range of 77 to 47,488). All of the measures that met eligibility criteria were published after 1996. Of the 15 measures, 11 were published within the last 10 years (since 2006). All measures collected responses via pen and paper questionnaires and were conducted within the school setting. Some measures were administered verbally to students who identified as having English as their second language.

**Table 4:** Characteristics of identified school connectedness measures and description of studies describing their development and validation.

Measure (Acronym); Published Year	Purpose*; description of measure	Number of subscales	Total items	Response options; time to complete	Reference	Study purpose	Sample characteristics Age (range [R]; Mean [M], Standard Deviation [SD], Not Reported [NR]).
**Perceived School Experiences Scale (PSES), 2012**	Descriptive, discriminative and predictive. For use by social workers to assess students’ perceptions of their school experience for school improvement planning.	3 SS: School Connectedness; Academic Press; Academic Motivation.	14	5 point Likert (1 –strongly disagree, 5 –strongly agree). 30 minutes.	Anderson-Butcher, Amorose, Iachini & Ball [62]	To develop and evaluate psychometric properties of the PSES.	N = 870. United States. Study 1 –exploratory and confirmatory factor analysis. Calibration sample (n = 386): Year of enrolment: Year 7 (8.5%), Year 8 (32%), Year 9 (8.8%); Year 10 (9.8%); Year 11 (10.95%), Year 12 (29.95%). Gender: Female (53.1%); Male (46.9%). Ethnicity: Caucasian (71%); African American (14%); Multi-racial (8.8%); Other (6.2%). Excluded findings from Study 2 (test retest reliability and hypothesis testing) as only had 3 of 97 participants meeting age criteria.
**Student Engagement in Schools Questionnaire (SESQ), 2008**	Descriptive and discriminative. Measures students perspectives of facilitators and indicators of engagement	5 SS: Affective—Liking for Learning; Affective—Liking for School; Behavioural—Effort and Persistence; Behavioural—Extra Curricular; Cognitive Engagement.	109	5 point Likert (1 –never, 5 –always). 35 minutes	Hart, Stewart & Jimerson [13]	To establish the psychometric properties of the SESQ.	N = 428. United States. Year of enrolment: Year 7 (36%); Year 8 (5%); Year 9 (59%). Gender: Male (54%); Female (46%). Ethnicity: Hispanic (42%); African American (25%); Caucasian (6%); Other (27%).
**Student Engagement Instrument (SEI), 35 item version, 2004**	Descriptive, discriminative and predictive. Measures students’ level of engagement as well as determination of goodness of fit between student and learning environment and factors that influence the fit.	6 SS: Teacher-Student Relationships; Control and Relevance of School Work; Peer Support for Learning; Future Aspirations and Goals; Family Support for Learning Extrinsic Motivation.	35	4 point Likert (1 –strongly disagree, 5 –strongly agree). 20 to 30 minutes.	Appleton, Christenson, Kim & Reschly [28]	To examine the psychometric properties of the SEI.	N = 1,931. United States. Year of enrolment: Year 9 (100%). Gender: Female (51%); Male (49%). Ethnicity: African American (40.4%); White (35.1%); Asian (10.8%); Hispanic (10.3%); American Indian (3.4%). Speak languages other than English (22.9%).
**Student Engagement Instrument (SEI), 33 item version, 2010**	See above.	5 SS: Teacher-Student Relationships; Control and Relevance of School Work; Peer Support for Learning; Future Aspirations and Goals; Family Support for Learning	33	4 point Likert (1 –strongly disagree, 5 –strongly agree). 20 to 30 minutes	Betts, Appleton, Reschly, Christenson & Huebner [60]	Examine the psychometric properties of the SEI.	N = 2416. United States. Two districts: South Carolina (n = 418) and Minnesota (n = 1998). Year of enrolment: Years 6 to 12 (300 students per grade). Gender: Males (n = 1197); Females (n = 1219). Ethnicity: European American (86%), African American (9%), Asian American (1%), Hispanic (2%), Native American (2%). Less than 2% indicated that English was second language.
					Reschly, Betts & Appleton [61]	Examine psychometrics of two measures of student engagement.	N = 277. United States. Year of enrolment: Year 9, 10 and 12 (mean age of 17 years) Gender: Female (57%); Males (43%). Ethnicity: African American (71%); Other (29%)
					Lovelace et al. [57]	Examine concurrent and predictive validity of the SEI.	N = 47,488. United States. Sample 1 –concurrent validity (n = 35, 900). Year of enrolment: Year 6 (33.6%); Year 7 (34.6%), Year 8 (31.8%). Gender: Female (48.5%); Male (51.5%). Ethnicity: Caucasian (35.1%); African American (22.8%), Hispanic (10.3%): Asian (4.1%), Multiracial (<1%): Other (26.7%). English speaking (68.5%); Spanish speaking (19/9%). Students receiving special education services (13.6%). Sample 2 –predictive validity (n = 11588). Gender: Female (49.8%); Male (50.2%). Ethnicity: Caucasian (37.4%); African American (26.5%), Hispanic (20.4%): Asian (10.5%), Multiracial (4.6%); Other (0.6%). English speaking (72.3%); Spanish speaking (15.5%). Students receiving special education services (10.9%).
**Student Engagement Instrument—Elementary Version, 2012**	See above	4 SS: Teacher Student Relationships Peer Support for Learning Future Goals and Aspirations Family Support for Learning	24	4 point Likert (1 –strongly disagree, 5 –strongly agree). 20 to 30 minutes	Carter et al. [55]	To validate the elementary version of the SEI.	N = 1,943. United States. Year of enrolment: Equivalent samples across Year 3 to 5. Gender: Equal male and female. Ethnicity: African American (29.8%); Hispanic (28.9%); Caucasian (28.6%); Asian / Pacific Islander (8.5%); Multi-racial (4.2%). Students receiving special education services (13.7%); English language learners (16.2%).
**Student Subjective Wellbeing Questionnaire (SSWQ), 2014**	Descriptive, discriminative and predictive. Measures students’ subjective wellbeing at school.	4 SS: Academic Efficacy Educational Purpose Joy of Learning School Connectedness	16	4 point Likert (1 –almost never, 5 –almost always)	Renshaw, Long, Cook [63]	To develop and validate the SSWQ.	N = 1,002. United States. Year of enrolment: Year 6 to 8 across two schools. Ethnicity (School Sample 1): African American (63%); Caucasian (26%); Multiple ethnicities (11%). Ethnicity (School Sample 2): African American (73%), Caucasian (13%); Multiple ethnicities (14%).
					Renshaw et al. [58]	Investigate latent factor structure, factor/scale characteristics, multi group measurement invariance and potential utility of the SSWQ.	N = 438. United States. Year of enrolment: Year 6 (49.1%) and Year 7 (50.9%). Ethnicity African American (63%); Caucasian (26%); Hispanic (5%); Asian or Pacific Islander (3%); Multiple ethnicities (3%). Eligible for free or reduced price lunch (76%); qualified for special education services (9%).
**Developmental School Climate Survey—Full Version, 2000**	Discriminative and evaluative. Assesses students perceptions of school climate	5 SS: School environment Academic attitudes and motives Personal attitudes, motives and feelings Social attitudes, motivates and behaviour Cognitive/ academic performance.	100	Not Reported	Solomon, Battistich, Watson, Schaps & Lewis [56]	To evaluate comprehensive elementary school program over a three-year period. Demonstrated factor structures and reliabilities within paper.	N = 4,373 to 5,011. United States. Year of enrolment: elementary schools over six districts from Year 3 to 6.
**Developmental School Climate Survey—Abbreviated Version, 2011**	See above	7 SS: Positive behaviour Negative behaviour Classroom and school supportiveness Autonomy and influence Safety at school Enjoyment of class / school liking School norms and rules	34	Not Reported	Ding, Liu & Berkowitz [59]	To examine the factor structure and reliability of an abbreviated version of the Developmental School Climate Survey	N = 6,500. United States. 24 elementary schools. Ethnicity: African American (58%), Caucasian (26%); Hispanic (13%), Other (3%). Students with special needs (27.3%).
**Student Personal Perception of Classroom Climate (SPPCC), 2010**	Descriptive; Measures students perceptions of classroom climate	4 SS: Teacher support Academic Competence Satisfaction Peer Support	26	4 point Likert (1 –never, 4 –almost always)	Rowe, Kim, Baker, Kamphaus & Horne [64]	To examine the factor structure of the SPPCC.	N = 589. United States. Study 1 –Sample (n = 267). Year of enrolment Year 3 (35%); Year 4 (32%); Year 5 (33%). Gender: Males (47%); Females (53%). Ethnicity: African American (46%); Caucasian (34%); Hispanic (7%); Asian Pacific (2%); Multiracial (2%), Other (8%). Study 2—Sample (n = 322). Year of enrolment: Year 3 (35%); Year 4 (32%); Year 5 (33%). Gender: Males (49%); Females (51%). Ethnicity: African American (29%); Caucasian (24%); Hispanic (9%); Asian / Pacific (2%); Multiracial (2%); Other (34%).
**Student Personal Perception of Classroom Climate (SPPCC), Adapted Version, 2016**	See above.	4 SS: Teacher support Academic Competence Satisfaction Peer Support	26	5 point Likert (1 –false, 5 –true)	Rubie Davies, Asil & Teo [54]	To assess measurement invariance of SPCC with NZ sample.	N = 1,924. New Zealand. Year of enrolment: Year 3 (5.7%); Year 4 (18.5%), Year 5 (18.5%), Year 6 (17.7%), Year 7 (19.2%); Year 8 (20.4%). Gender: Female (49.9%); Male (50.1%). Ethnicity: New Zealand European (47%), Maori (18.8%); Pacific Islander (16.3%), Asian (14.8%); Other (3.1%)
**Identification with School Questionnaire, 1996**	Descriptive and discriminative. Measures students’ identification with school.	2 SS: Belongingness in school Feelings of valuing school and school related outcomes	16	4 point Likert (1 –strongly agree, 4 –strongly disagree)	Voekl [65]	To develop and validate the Identification with School Questionnaire.	N = 3,539. United States. Year of enrolment: Year 8 students. Gender: Male (M = 48.38; SD = 6.76); Female (M = 50.66; SD: 5.78).
**Student School Engagement Survey (SSES), 2006**	Descriptive, discriminative and predictive. Measures students level of engagement in three domains	3 SS: Emotional engagement Cognitive engagement Behavioural engagement	45	Likert scale (strongly agree to strongly disagree)	National Centre for School Engagement [39]	To develop and validate the SSES.	N = 135. United States. Year of enrolment: Elementary school students, age (M/SD/R = NR)
**School Bonding Index Revised (SBI-R), 2003**	Descriptive, discriminative and predictive. Measures youth level of attachment to and comfort with school.	4 SS: School experience School involvement School delinquency School pride	24	Likert scale	Rodney, Johnson & Srivastava [66]	To evaluate effectiveness of the Family and Community Violence Prevention Program on youth violence; reports on psychometrics of SBI-R.	N = 2,548. United States. Year of enrolment: under age of 12 (28.5%); over age of 12. Gender: Male (58%); Female (42%). Ethnicity: African Americans (72%); Hispanics (10.3%). Native Americans and Native Hawaiians (15%); Other (2.7%).
**School Climate Measure (SCM), 2010**	Descriptive, discriminative and predictive. Measures students perceptions of school climate	8 SS: Positive Student-Teacher Relationships School Connectedness Academic Support Order and Discipline School Physical Environment School Social Environment Perceived Exclusion Privilege Academic Satisfaction	39	5 point Likert (1 –strongly disagree, 5 –strongly agree)	Zullig, Koopman, Patton & Ubbes [67]	To develop and validate the SCM.	N = 21,082. United States. Year of enrolment: Year 6 (14.4%); Year 7 (16.1%); Year 8 (14.7%); Year 9 (16.8%), Year 10 (15.8%), Year 11 (10.9%), Year 12 (11.3%). Gender: Males (50.1%); Females (49.9%); Ethnicity: White and Non Hispanic (84%); Other (5.4%); African American (2.3%), Asian (2.2%); American Indian or Alaskan Native (6.1%).
					Zullig, Collins, Ghani, Patton, Huebner & Ajamie [68]	To further validate SCM on four domains (positive-student teacher relationships, academic support, order and discipline and physical environment)	N = 10,253. United States. Year of enrolment: 14 years or younger (7.38%); older than 14 years (92.62%). Gender: Males (48.93%). Females (51.07%). Ethnicity: Hispanic (48.6%); Caucasian (36.1%); American Indian or Alaskan Native (4.9%), Native Hawaiian or Other Pacific Islander (1.4%); African American (6.2%), Asian (2.8%).
					Zullig, Collins, Ghani, Hunter, Patton, Huebner & Zhang [69]	To further validate the SCM on larger sample before the addition of two new domains (see below).	N = 1,643. United States. Year of enrolment: Year 9 (22.3%); Year 10 (19%), Year 11 (40.9%), Year 12 (17.8%). Gender: Males (49.6%). Females (50.4%). Ethnicity: Hispanic or Latino (61.2%), White Non-Hispanic (18.5%); African American (6.8%); Other (13.5%).
**School Climate Measure (SCM)–Revised Version, 2015**	See above.	10 SS: Positive Student-Teacher Relationships School Connectedness Academic Support Order and Discipline School Physical Environment School Social Environment Perceived Exclusion Privilege Academic Satisfaction Parental involvement Opportunities for student engagement	42	5 point Likert (1 –strongly disagree, 5 –strongly agree)	Zullig, Collins, Ghani, Hunter, Patton, Huebner & Zhang [69]	To further validate the SCM on larger sample with two new domains (parental involvement and opportunities for student engagement)	N = 1,643. United States. Year of enrolment: Year 9 (22.3%); Year 10 (19%), Year 11 (40.9%), Year 12 (17.8%). Gender: Males (49.6%). Females (50.4%). Ethnicity: Hispanic or Latino (61.2%), White Non-Hispanic (18.5%); African American (6.8%); Other (13.5%).

Notes.

* Purpose of measures: descriptive (i.e. describes current status, problems, needs and/or circumstances); discriminative (i.e. distinguishes between individuals or groups on a characteristic or underlying dimension); predictive (i.e. classifies individuals into pre-defined categories of interest), evaluative (i.e. detects magnitude of change over time within one person or a group of people after intervention).

Refer to S1 File for further information about excluded publications and reasons for exclusion.

Table 5 summarises the domains of school connectedness measured by each instrument. The subdomains were categorised following a thematic synthesis by four members of the research team based on the definitions or descriptions of the scales and/or subscales in included studies. Subdomains were identified and subsumed under the most relevant domain: (1) affective (i.e., feelings of acceptance, belonging and inclusion; feelings of respect and being respected; value importance of school; feelings of safety; sense of autonomy and independence and academic self-efficacy), (2) cognitive (i.e., perceptions of—teacher relationships and support; peer relationships and support; academic support; discipline, order and fairness; and the value parents place on school) and (3) behavioural (i.e., involvement, participation and engagement; effort and persistence; conduct and interest and motivation). No single instrument measured all aspects of affective, cognitive and behavioural domains of school connectedness. The measure that measured the most aspects was versions of the Student Engagement Instrument (i.e., 35 item, 33 item and elementary version) [36, 55, 57, 60, 61], which measured 12 of 15 affective, cognitive and behavioural components of school connectedness.

**Table 5:** Domains and concepts of school connectedness measured by included instrument.

	Affective					Cognitive					Behavioural				
Measure	1	2	3	4	5	6	7	8	9	10	11	12	13	14	15
**PSES**	X		X			X	X		X			X			X
**SESQ**			X			X	X	X	X		X	X	X	X	X
**SEI 35 item**		X	X	X	X	X	X	X		X	X	X	X		X
**SEI 33 item**		X	X	X	X	X	X	X		X	X	X	X		X
**SEI—E**		X	X	X	X	X	X	X		X	X	X	X		X
**SSWQ**	X	X	X			X						X			X
**Developmental School Climate Survey**		X	X	X	X				X	X				X	
**Developmental School Climate Survey—Abbreviated**		X	X	X	X				X	X				X	
**SPPCC**	X					X	X	X	X			X			X
**SPPCC—Adapted**	X					X	X	X	X			X			X
**Identification with School**		X	X				X	X				X			
**SSES**		X	X			X				X		X		X	X
**SBI-R**	X		X		X		X					X		X	
**School Climate Measure**			X		X	X	X		X	X			X		X
**School Climate Measure—Revised**			X		X	X	X		X	X	X		X		X

Note.

^1^Acceptance, Inclusion and Belonging;

^2^ Respect;

^3^ Value;

^4^ Safety;

^5^Autonomy and Independence;

^6^Academic Self Efficacy;

^7^Teacher Relations & Support;

^8^Peer Relations & Support;

^9^Academic Support;

^10^Discipline, fairness and order;

^11^Value parents place on school;

^12^Involvement, participation and engagement;

^13^Effort and persistence;

^14^Conduct;

^15^Interest or motivation.

### Psychometric properties

Table 6 summarises quality ratings of psychometric studies and therefore risk of bias as determined by COSMIN. All measures included in the review were found to have good to excellent study quality for internal consistency, structural validity and hypothesis testing and poor to excellent study quality for content validity. Internal consistency and structural validity were the most frequently reported properties having being described in 17 and 16 studies respectively. Content validity was described for eight measures and hypothesis testing for 10 measures. Five studies reporting on hypothesis testing, described findings for more than one hypothesis. Of the 15 included instruments, six were revisions of earlier versions of measures of school connectedness (i.e., SEI– 35 item [36], SEI– 33 item [57, 60, 61], SEI—Elementary [55], Developmental Study Centre’s School Climate Survey—Abbreviated Version [59], SPPCC—Adapted [54], SCM—Adapted [69]). These measures were evaluated separately as the item pool and response format of these measures had been changed. For 11 measures only single studies were identified. The SEI (33 item version) [57, 60, 61] and the SCM [67, 68] had the most studies; reporting on psychometric properties in three research articles. Thirteen measures reported on two or more of six psychometric properties (average 3; range 1–4). The PSES [62] and the Developmental Study Centre’s School Climate Survey (Full Version) [56] were the only measures to report on one psychometric property. Many measures had no published information relating to content validity including the PSES [62], SESQ [13], SEI– 33 item version [57, 60, 61], Developmental Study Centre’s School Climate Survey (Full Version and Abbreviated Version) [56, 59], SBI—R and SCM (Revised Version). The only study that was excluded from further analysis in the review was by Voekl [65] for receiving a poor COSMIN rating for content validity.

**Table 6:** Overview of the psychometric properties and methodological quality of school connectedness measures.

Measure & Author(s)	Internal Consistency	Reliability	Measurement Error	Content Validity	Structural Validity	Hypothesis testing
**PSES**						
**Anderson-Butcher, Amorose, Iachini & Ball** [62]	NR	NR	NR	NR	Good (75.0)	NR
**SESQ**						
**Hart, Stewart & Jimerson** [13]	Excellent (85.7)	NR	NR	NR	Good (75.0)	Good (65.2)
**SEI– 35 item version**						
**Appleton, Christenson, Kim & Reschly** [28]	Excellent (85.7)	NR	NR	Excellent (78.6)	Excellent (100.0)	Good (52.2)
**SEI– 33 item version**						
**Betts, Appleton, Reschly, Christenson & Huebner** [60]	NR	NR	NR	NR	Good (75.0)	NR
**Reschly, Betts & Appleton** [61]	Excellent (90.5)	NR	NR	NR	Good (66.7)	Excellent (91.3)
						Excellent (91.3)
						Excellent (87.0)
						Excellent (73.9)
						Good (69.6)
**Lovelace, Reschly, Appleton & Lutz** [57]	NR	NR	NR	NR	NR	Excellent (94.1)
						Excellent (94.1)
						Excellent (87.0)
						Excellent (94.1)
**SEI—E**						
**Carter et al.** [55]	Excellent (100)	NR	NR	Excellent (78.6)	Excellent (100)	Excellent (76.5)
						Excellent (76.5)
**SSWQ**						
**Renshaw, Long, Cook** [63]	Excellent (100)	NR	NR	Excellent (100)	Excellent (100)	Excellent (87.0)
						Excellent (87.0)
						Excellent (87.0)
**Renshaw et al.** [58]	Excellent (85.7)	NR	NR	NR	Excellent (100)	Good (65.2)
**Developmental School Climate Survey—Full Version**						
**Solomon, Battistich, Watson, Schaps & Lewis** [56]	Good (52.4)	NR	NR	NR	NR	NR
**Developmental School Climate Survey—Abbreviated Version**						
**Ding, Liu & Berkowitz** [59]	Excellent (85.7)	NR	NR	NR	Good (58.3)	NR
**SPPCC**						
**Rowe, Kim, Baker, Kamphaus & Horne** [64]	Excellent (85.7)	NR	NR	Fair (42.9)	Excellent (91.7)	NR
**SPPCC—Adapted Version**						
**Rubie Davies, Asil & Teo** [54]	Excellent (76.2)	NR	NR	Good (57.1)	Excellent (100)	Excellent (76.5)
**Identification with School Questionnaire**						
**Voekl** [65]	Excellent (85.7)	NR	NR	Poor (21.4)	Good (75.0)	Good (58.8)
**SSES**						
**National Centre for School Engagement** [39]	Good (57.1)	NR	NR	Good (57.1)	NR	Good (52.2)
						Good (64.7)
**SBI—R**						
**Rodney, Johnson & Srivastava** [66]	Good (66.7)	NR	NR	NR	NR	Good (65.2)
**SCM**						
**Zullig, Koopman, Patton & Ubbes** [67]	Excellent (85.7)	NR	NR	Excellent (92.9)	Good (75.0)	NR
**Zullig, Collins, Ghani, Patton, Huebner & Ajamie** [68]	Excellent (100)	NR	NR	NR	Excellent (100)	Excellent (82.6)
**Zullig, Collins, Ghani, Hunter, Patton, Huebner & Zhang** [69]	Excellent (85.7)	NR	NR	NR	Good (75.0)	NR
**SCM—Revised**						
**Zullig, Collins, Ghani, Hunter, Patton, Huebner & Zhang** [69]	Excellent (85.7)	NR	NR	NR	Good (75.0)	NR

Notes. The quality of the studies that evaluated the psychometric properties of each instrument was evaluated according to the COSMIN rating per item: four-point scale was used (1 = Poor, 2 = Fair, 3 = Good, 4 = Excellent). The overall methodological quality per study was presented as percentage of rating (Poor = 0–25.0%, Fair = 25.1%–50.0%, Good = 50.1%–75.0%, Excellent = 75.1%–100.0%). NR: not reported.

Refer to Table 7 for a summary of the quality of psychometric properties of included measures based on Terwee et al. [51] and Schellingerhout et al. (2012). Refer to Table 8 for a summary of the overall psychometric quality ratings per psychometric property for each measure as evaluated against Schellingerhout et al [52] criteria. A description of the criteria used to rate overall psychometric quality can be found in the notes section of Table 8.

**Table 7:** Quality of psychometric properties based on the criteria by Terwee et al. [51] and Schellingerhout [52].

Measure & author(s)	Internal consistency	Reliability	Measurement error	Content validity	Structural validity	Hypothesis testing
**PSES**						
Anderson-Butcher, Amorose, Iachini & Ball [62]	NR	NR	NR	NR	+	NR
**SESQ**						
Hart, Stewart & Jimerson [13]	-	NR	NR	NR	+	?
**SEI– 35 item version**						
Appleton, Christenson, Kim & Reschly [28]	+	NR	NR	+	?	?
**SEI– 33 item version**						
Betts, Appleton, Reschly, Christenson & Huebner [60]	NR	NR	NR	NR	?	NR
Reschly, Betts & Appleton [61]	+	NR	NR	NR	?	+
Lovelace, Reschly, Appleton & Lutz [57]	NR	NR	NR	NR	NR	+
**SEI—E**						
Carter et al. [55]	-	NR	NR	+	?	?
**SSWQ**						
Renshaw, Long & Cook [63]	+	NR	NR	+	+	+
Renshaw et al. [58]	?	NR	NR	NR	?	?
**Developmental School Climate Survey—Full Version**						
Solomon, Battistich, Watson, Schaps & Lewis [56]	?	NR	NR	NR	NR	NR
**Developmental School Climate Survey—Abbreviated Version**						
Ding, Liu & Berkowitz [59]	-	NR	NR	NR	?	NR
**SPPCC**						
Rowe, Kim, Baker, Kamphaus & Horne [64]	-	NR	NR	±	-	NR
**SPPCC—Adapted Version**						
Rubie Davies, Asil & Teo [54]	?	NR	NR	±	?	?
**Identification with School Questionnaire**						
Voekl [65]	+	NR	NR	NE	?	?
**SSES**						
National Centre for School Engagement [39]	+	NR	NR	±	NR	+
**SBI—R**						
Rodney, Johnson & Srivastava [66]	?	NR	NR	NR	NR	?
**SCM**						
Zullig, Koopman, Patton & Ubbes [67]	+	NR	NR	+	-	NR
Zullig, Collins, Ghani, Patton, Huebner & Ajamie [68]	+	NR	NR	NR	+	+
Zullig, Collins, Ghani, Hunter, Patton, Huebner & Zhang [69]	-	NR	NR	NR	+	NR
**SCM—Revised**						
Zullig, Collins, Ghani, Hunter, Patton, Huebner & Zhang [69]	-	NR	NR	NR	+	NR

Notes. Quality criteria: + = positive rating;? = indeterminate rating;- = negative rating; ± = conflicting data; NR = not reported; NE = not evaluated (study of poor methodological quality according to COSMIN rating—data are excluded from further analyses).

**Table 8:** Overall quality score of assessments for each psychometric property based on levels of evidence by Schellingerhout et al. [52].

Measure	Internal consistency	Reliability	Measurement error	Content validity	Structural validity	Hypothesis testing
**PSES**	NR	NR	NR	NR	Moderate (positive)	NR
**SESQ**	Strong (negative)	NR	NR	NR	Moderate (positive)	Indeterminate
**SEI– 35 item**	Strong (positive)	NR	NR	Strong (positive)	Indeterminate	Indeterminate
**SEI– 33 item**	Strong (positive)	NR	NR	NR	Indeterminate	Strong (positive)
**SEI—E**	Strong (negative)	NR	NR	Strong (positive)	Indeterminate	Indeterminate
**SSWQ**	Indeterminate	NR	NR	Strong (positive)	Indeterminate	Indeterminate
**Developmental School Climate Survey—Full Version**	Indeterminate	NR	NR	NR	NR	NR
**Developmental School Climate Survey—Abbreviated Version.**	Strong (negative)	NR	NR	NR	Indeterminate	NR
**SPPCC**	Strong (negative)	NR	NR	Conflicting	Strong (negative)	NR
**SPPCC—Adapted Version**	Indeterminate	NR	NR	Conflicting	Indeterminate	Indeterminate
**Identification with School Questionnaire**	Strong (positive)	NR	NR	NE	Indeterminate	Indeterminate
**SSES**	Moderate (positive)	NR	NR	Conflicting	NR	Strong (positive)
**SBI—R**	Indeterminate	NR	NR	NR	NR	Indeterminate
**SCM**	Moderate (positive)	NR	NR	Strong (positive)	Conflicting	Strong (positive)
**SCM—Revised**	Strong (negative)	NR	NR	NR	Moderate (positive)	NR

Notes. Levels of Evidence: Strong evidence positive/negative result = Consistent findings in multiple studies of good methodological quality OR in one study of excellent methodological quality; Moderate evidence positive/negative result = Consistent findings in multiples studies of fair methodological quality OR in one study of good methodological quality; Limited evidence positive/negative = One study of fair methodological quality; Conflicting findings; Indeterminate = only indeterminate measurement property ratings (i.e., score = ? in Table 7); NR = Not reported; Not Evaluated = studies of poor methodological quality according to COSMIN excluded from further analyses.

## Discussion

There is no universally accepted definition of school connectedness; however, the construct is referred to regularly within the literature and is a key area in informing educational policy and reform [39]. The reliable and valid measurement of school connectedness is important to researchers and educators, to minimise the long term documented implications of reduced school connectedness on students’ academic success and socio-emotional wellbeing through the provision of appropriate school based supports. This systematic review provides a comprehensive summary of the quality of psychometric properties of self-report school connectedness measures available for students aged 6 to 14 years using the COSMIN taxonomy of measurement properties.

### Quality of the studies using the COSMIN taxonomy

Construct validity, within the COSMIN taxonomy, comprises structural validity, hypothesis testing and content validity [43]. To confidently select and use measures in research it is important to understand “…how well [the] measure assesses what it claims to measure and how well it holds its meaning across varied contexts and sample groups” [45]. Construct validity supersedes all other psychometric properties in measurement development as it is irrelevant if an instrument has good reliability if the construct which it measures is not well established. Many instruments are currently being used to assess school connectedness or related terms. Interestingly, however, the majority of studies in this review failed to adequately define or conceptualise the construct of school connectedness. Rather, studies focused on describing the methodology they used to develop the measure, including the statistical analyses used to test psychometric properties.

A lack of conceptualisation of school connectedness has made it difficult to: (a) adequately compare measures in this review; (b) determine if included measures fully operationalise the construct of school connectedness; and (c) determine whether students sense of school connectedness has changed, or whether change is due to the evolving nature of the construct and the way it is understood currently by researchers and educators in the field. As illustrated in Table 5, none of the measures included in this review, fully capture all aspects of school connectedness and in addition, the quality of descriptions were lacking.

The majority of studies included in this review fail to explicitly state the intended purpose of the measure. That is, whether the instrument was originally intended as an outcome measure to evaluate changes over time following the implementation of school based supports or whether it was intended purely as a diagnostic tool to identify whether school based supports are required. Without this information, researchers and educators may make inappropriate choices and misinterpret assessment findings; leading to errors in clinical judgement. Future research should focus on developing a universal definition of school connectedness and further validate included measures.

Test-retest, inter-rater and intra-rater reliability and measurement error were not reported for any measures included in this review. Given that psychological constructs, such as school connectedness, are relatively stable over time it is important to utilise measures that have low error and are able to detect minor changes over time. Preliminary reliability testing is necessary to evaluate an instruments responsiveness. Without this information, it is difficult to make evidence based informed choices when selecting measures in research. This being said, some measures included in the review such as the SSES [39] have been used in research to evaluate changes in school connectedness over time. Although responsiveness was not evaluated in this review, researchers and educators should exercise caution when using included measures due to a lack of information on their reliability.

Some studies included in the review reported verbal administration of measures to students who identified as using English as their second language. This method of administration places a high demand on students’ expressive and receptive language skills as well as their verbal comprehension and memory recall resulting in a potential for error in the recorded true scores. Minor changes in question wording, question order or response format can result in different findings [40]. This method of questionnaire administration may have impacted the quality of findings in these studies. Furthermore, it is important to consider inherent bias that exists with self-report measures. Student responses may be affected by their perception of support within their school–“…they may take into account social norms when responding, which may result in social desirability bias” [40]. Methods do exist to reduce this problem such as assuring students of confidentiality and anonymity; however, this can increase students suspicions about the sensitivity of the topic [40]. Many studies included in the review failed to explicitly state how measures were administered and/or did not report on efforts to minimise the impact of social desirability bias on data quality.

Although the focus of this review was to evaluate the psychometric properties of school connectedness measures for students aged 6 to 14 years, the samples of included studies largely comprised older students up to the age of 18 years. Students under the age of 12 years represented approximately 25% of samples in included studies. This calls into question the utility and appropriateness of these measures with younger student populations. When examining included measures in more detail, it was noted many measures had lengthy item pools. For example, the Developmental Study Centre’s School Climate Survey (Full Version) [56] and the SESQ [13] included 100 and 109 items respectively. Not only would these measures be time consuming, they would require a great deal of concentration for a young student to complete. It is important to be able to validly and reliably assess students’ sense of school connectedness in early primary school in order to identify and support at-risk students to prevent the long-term documented implications of a lack of school connectedness on student outcomes. Future research should focus on validating included measures with younger students to ensure measures are age appropriate and can be reliably and validly used in this population.

### Overall quality of psychometric properties

The overall quality of measurement properties critiqued in this study varied widely. The school connectedness self-report measures with the strongest psychometric properties were the SCM [67–69] and the 35–item version of the SEI [36]. The SCM [67–69] addressed eight of 15 school connectedness components (see Table 5) and reported on four of six psychometric properties (see Table 6); scoring strong positive ratings for content validity and hypothesis testing, a moderate positive rating for internal consistency and a conflicting rating for structural validity. The 35–item version of the SEI [36] reported on four of six psychometric properties; scoring strong positive ratings for internal consistency and content validity and indeterminate ratings for structural validity and hypothesis testing. Interestingly, however, the SEI [36] addressed the most (i.e., 12 of 15) school connectedness components of any measure included in the review; suggesting that the SEI [36] not only has promising psychometrics but encompasses a broader range of school connectedness components. The school connectedness measure with the poorest psychometric properties was the SPPCC [54], reporting on three of six psychometric properties; scoring strong negative ratings for internal consistency and structural validity, and conflicting results for content validity. Across all measures and measurement properties there were a number of conflicting ratings (14%), many indeterminate ratings (41%), and missing data (36%); suggesting more research is required to determine the psychometric qualities of these measures.

An in-depth discussion about the statistical frameworks used in included articles is outside the scope of this review; however, it is noteworthy to draw reader’s attention to the fact that none of the measures included in this review were tested at an item level using IRT. All measures were tested using CTT. A major limitation of CTT is its relatively weak theoretical assumptions and circular dependency; that is “(a) the person statistic (i.e., observed score) is (item) sample dependent and (b) the item statistics are (examinee) sample dependent; which poses some difficulties in CTT’s application in some measurement situations” [70]. IRT was developed to address the main limitations of CTT. However, IRT does have its own limitations in that it is a complex model requiring much larger samples of participants compared to CTT [71]. Even with the need for larger samples when using IRT, the benefits of IRT outweigh the singular use of CTT [70, 71]. IRT assists in determining whether (a) a measure has any redundant items; (b) items are functioning sufficiently to adequately capture the construct of interest; and (c) the response format is operating appropriately [70]. Future research should test included measures using IRT to gain a more in-depth understanding of measures functioning at an item level.

### Limitations

Although every effort was taken to ensure the scientific rigor of this systematic review, there were a number of limitations. Information published in languages other than English were not included. Therefore, there may be some relevant findings regarding the psychometric properties of measures that were not included in this review. In addition, authors of included studies were not contacted therefore some information may have been overlooked. Furthermore, evaluating the quality of criterion validity, cross cultural validity and responsiveness was outside the scope of this review.

## Conclusion

As school connectedness is both a precursor to and an outcome of academic success, it is important to be able to reliably and validly assess students’ sense of school connectedness in order to accurately identify and support at-risk students [17, 39]. The current systematic review reported on the psychometric properties of 15 self-report school connectedness measures for students aged between 6 and 14 years of age. The measures with the strongest psychometric properties was the SCM and the 35**–**item version SEI exploring 8 and twelve (of 15) school connectedness components respectively. This systematic review highlighted the need for further research to examine the psychometric properties of existing school connectedness measures that were identified as having moderate to strong positive evidence.

## Supporting information

S1 Table PRISMA 2009 checklist. (DOCX)

S2 Table Search terms. (DOCX)

S3 Table Overview of school connectedness instruments: Reasons for exclusion. (DOCX)

S1 File Excluded publications and reasons for exclusion. (DOCX)
