# Supplementary material for: Evaluating the psychometric quality of school connectedness measures: A systematic review
Source: PLoS One. 2018 Sep 11;13(9):e0203373. doi: 10.1371/journal.pone.0203373 (PMC6133283; doi:10.1371/journal.pone.0203373)
Supplement: S2 Table — (DOCX) [file pone.0203373.s002.docx]

**S2 Table. Search terms**

|  | **Initial search: Assessment retrieval**  **Database and Search Terms (Subject Headings and Free Text Words)** | **Limits** | **No. of records** |
| --- | --- | --- | --- |
| **Subject**  **Headings** | **CINAHL**: ((MH "Students, High School") OR (MH "Students") OR (MH "Students, Middle School") OR (MH "Students, Elementary") OR (MH "Adolescence") OR (MH "Child") OR (MH "Schools, Middle") OR (MH "Schools, Secondary") OR (MH "Schools, Elementary") OR (MH "Schools") OR (MH "Child, Preschool") OR (MH "Early Intervention") OR (MH "Early Childhood Intervention") OR (MH "Education")) AND ((MH "Social Inclusion") OR (MH "Social Participation") OR (MH "Social Adjustment") OR (MH "Social Attitudes") OR (MH "Membership") OR (MH "Commitment") OR (MH "Social Involvement (Iowa NOC)") OR (MH "Social Inclusion") OR (MH "Student Experiences") OR (MH "Social Participation") OR (MH "Student Attitudes") OR (MH "Social Adjustment"))AND ((MH "Outcome Assessment") OR (MH "Patient Assessment") OR (MH "Self Assessment") OR (MH "Psychological Tests") OR (MH "Research Measurement") OR (MH "Scales") OR (MH "Questionnaires") OR (MH "Research Instruments") OR (MH "Treatment Outcomes") OR (MH "Evaluation") OR (MH "Evaluation Research") OR (MH "Self Assessment") OR (MH "Patient Assessment")) AND ((MH "Psychometrics") OR (MH "Measurement Issues and Assessments") OR (MH "Validity") OR (MH "Predictive Validity") OR (MH "Reliability and Validity") OR (MH "Internal Validity") OR (MH "Face Validity") OR (MH "External Validity") OR (MH "Discriminant Validity") OR (MH "Criterion-Related Validity") OR (MH "Consensual Validity") OR (MH "Concurrent Validity") OR (MH "Qualitative Validity") OR (MH "Construct Validity") OR (MH "Content Validity") OR (MH "Instrument Validation") OR (MH "Validation Studies") OR (MH "Test-Retest Reliability") OR (MH "Sensitivity and Specificity") OR (MH "Reproducibility of Results") OR (MH "Reliability") OR (MH "Intrarater Reliability") OR (MH "Interrater Reliability") OR (MH "Measurement Error") OR (MH "Bias (Research)") OR (MH "Selection Bias") OR (MH "Sampling Bias") OR (MH "Precision") OR (MH "Sample Size Determination") OR (MH "Repeated Measures")) | NA | 486 |
|  | **Embase:** (Student/ OR Adolescent/ OR Adolescence/ OR Child/ OR Juvenile/ OR School/ OR Preschool child/ OR early intervention/ OR Education/) AND (emotional attachment/ OR social environment/ OR Experience/ OR Attitude/ OR Adjustment/) AND (measurement/ or diagnostic procedure/ or rating scale/ or screening/ or screening test/ or questionnaire/ or outcome assessment/ or evaluation study/) AND (psychometry/ or validity/ or reliability/ or measurement error/ or measurement precision/ or measurement repeatability/ or error/ or statistical bias/ or test retest reliability/ or intrarater reliability/ or interrater reliability/ or accuracy/ or criterion validity/ or internal validity/ or face validity/ or external validity/ or discriminant validity/ or concurrent validity/ or qualitative validity/ or construct validity/ or content validity/) | NA | 454 |
|  | **ERIC:** (DE "Students" OR DE "High School Students" OR DE "Secondary School Students" OR DE "Middle School Students" OR DE "Junior High School Students" OR DE "Elementary School Students" OR DE "Classes (Groups of Students)") OR DE "Late Adolescents" OR DE "Early Adolescents" OR DE "Adolescents" OR DE "Children" OR DE "Youth" OR DE "Preschool Education" OR DE "Preschool Children" OR DE "Early Intervention" OR DE "Kindergarten" OR DE "Preschool Children" OR DE "Early Childhood Education" OR DE "Elementary Secondary Education" OR DE "Educational Environment" OR DE "Educational Experience" OR DE "Schools" OR DE "Primary Education" OR DE "Elementary Schools") AND (DE "Group Membership" OR DE "Group Experience" OR DE "Learner Engagement" OR DE "Educational Environment" OR DE "Classroom Environment" OR DE "School Community Relationship" OR DE "School Involvement" OR DE "Student Participation" OR DE "Peer Acceptance" OR DE "Inclusion" OR DE "Early Experience" OR DE "Educational Experience" OR DE "Group Experience" OR DE "Learning Experience" OR DE "Social Experience" OR DE "Student Experience" OR DE "School Involvement" OR DE "Student Participation" OR DE "Student Attitudes" OR DE "School Attitudes" OR DE "Student Adjustment" OR DE “Student School Relationship”) AND (DE "Evaluation" OR DE "Evaluation Methods" OR DE "Measurement" OR DE "Measurement Instruments (1966 1980)" OR DE "Measurement Techniques" OR DE "Testing" OR DE "Tests" OR DE "Rating Scales" OR DE "Screening Tests" OR DE "Questionnaires" OR DE "Outcome Measures" OR DE "Evaluation" OR DE "Evaluation Methods" OR DE “Measures (Individuals)”) AND (DE "Psychometrics" OR DE "Validity" OR DE "Reliability" OR DE "Error of Measurement" OR DE "Bias" OR DE "Interrater Reliability" OR DE "Accuracy" OR DE "Predictive Validity" OR DE "Construct Validity" OR DE "Content Validity") | NA | 603 |
|  | **Medline:** (Students/ OR Adolescent/ OR Child/ OR Schools/ OR "Early Intervention (Education)"/ OR Education/) AND ((school.ti OR school.ab.) AND ((connectedness OR belonging* OR membership* OR bond*OR attachment* OR engage* OR climate* OR communit* OR affiliat* OR commitment* OR involve* OR disconnect* OR accept* OR experience* OR pride* OR value* OR inclusion* OR participat* OR orientat*).ti. OR (connectedness OR belonging* OR membership* OR bond*OR attachment* OR engage* OR climate* OR communit* OR affiliat* OR commitment* OR involve* OR disconnect* OR accept* OR experience* OR pride* OR value* OR inclusion* OR participat* OR orientat*).ab.)) AND (measurement/ or diagnostic procedure/ or rating scale/ or screening/ or screening test/ or questionnaire/ or outcome assessment/ or evaluation study/) AND (psychometrics/ OR "Bias (Epidemiology)"/) | NA | 428 |
|  | **PsycINFO**: (DE "Classmates" OR DE "Elementary School Students" OR DE "High School Students" OR DE "Junior High School Students" OR DE "Kindergarten Students" OR DE "Preschool Students" OR DE "Kindergartens" OR DE "Classroom Environment" OR DE "Schools" OR DE "Early Intervention" OR DE "Elementary Education" OR DE "High School Education" OR DE "Middle School Education" OR DE "Preschool Education" OR DE "Private School Education" OR DE "Public School Education" OR DE "Secondary Education" OR DE "School Adjustment" OR DE "School Environment") AND (DE "Belonging" OR DE "Membership" OR DE "Attachment Behaviour" OR DE "Student Engagement" OR DE "Psychological Engagement" OR DE "School Environment" OR DE "Classroom Environment" OR DE "Sense of Community" OR DE "Community Attitudes" OR DE "Affiliation Motivation" OR DE "Commitment" OR DE "Involvement" OR DE "Group Participation" OR DE "Social Acceptance" OR DE "Mainstreaming (Educational)" OR DE "Emotional States" OR DE "Participation" OR DE "Group Participation" OR DE "Adolescent Attitudes" OR DE "Child Attitudes" OR DE "Student Attitudes" OR DE "Emotional Adjustment" OR DE "School Adjustment" OR DE "Social Adjustment") AND (DE "Measurement" OR DE "Testing Methods" OR DE "Test Scores" OR DE "Scaling (Testing)" OR DE "Rating Scales" OR DE "Screening" OR DE "Screening Tests" OR DE "Questionnaires" OR DE "Evaluation") AND (DE "Psychometrics" OR DE "Statistical Validity" OR DE "Test Validity" OR DE "Statistical Reliability" OR DE "Test Reliability" OR DE "Error of Measurement" OR DE "Errors" OR DE "Response Bias" OR DE "Interrater Reliability" OR DE "Repeated Measures") | NA | 174 |
| **Free Text** | **CINAHL:** (student* OR adolescen* OR pupil* OR teen* OR child* OR learner* OR youth* OR juvenile* OR school* OR class* OR preschool* OR pre-school* OR (early AND intervention*) OR kindergarten* OR education*) AND (TI school OR AB school) AND (TI (connectedness OR belonging* OR membership* OR bond*OR attachment* OR engage* OR climate* OR communit* OR affiliat* OR commitment* OR involve* OR disconnect* OR accept* OR experience* OR pride* OR value* OR inclusion* OR participat* OR orientat*) OR AB (connectedness OR belonging* OR membership* OR bond*OR attachment* OR engage* OR climate* OR communit* OR affiliat* OR commitment* OR involve* OR disconnect* OR accept* OR experience* OR pride* OR value* OR inclusion* OR participat* OR orientat*)) AND (assessment* OR measure* OR questionnaire* OR test OR tests OR scale* OR screening* OR evaluation* OR questionnaire* OR evaluation*) AND (psychometric* OR reliability OR validit* OR reproducibility OR bias OR responsiveness) | Publication date: 01/06/2015 – 13/06/2016 | 52 |
|  | **Embase*:*** *As per CINAHL free text* | Publication date: ‘2015-Current’ | 411 |
|  | **ERIC:** *As per CINAHL free text* | Publication date: 01/06/2015 – 13/06/2016 | 95 |
|  | **Medline:** *As per CINAHL free text* | Publication date: ‘2015-Current’ | 442 |
|  | **PsycINFO:** *As per CINAHL free text* | Publication date: 01/06/2015 – 13/06/2016 | 306 |
