# Supplementary material for: Evaluating the psychometric quality of school connectedness measures: A systematic review
Source: PLoS One. 2018 Sep 11;13(9):e0203373. doi: 10.1371/journal.pone.0203373 (PMC6133283; doi:10.1371/journal.pone.0203373)
Supplement: S3 Table — (DOCX) [file pone.0203373.s003.docx]

**S3 Table. Overview of school connectedness instruments: Reasons for exclusion**

| Assessment name | Abbreviation | Reason for exclusion |
| --- | --- | --- |
| Psychological Sense of School Membership Scale [26] | PSSMS | Not a measure of school connectedness  (did not address behavioural domain) |
| Psychological Sense of School Membership Scale – Brief [75] | N/A | Not a measure of school connectedness  (did not address behavioural domain) |
| What’s Happening In This School – 49 items [76] | WHITS | Not a measure of school connectedness  (did not address behavioural domain; validated only with high school students) |
| What’s Happening In This Class – 70 items [77] | WIHIC | Validated with high school sample only |
| What’s Happening In This Class – 56 items [77] | WIHIC | Validated with high school sample only |
| What’s Happening In This Class – 20 items [77] | WIHIC | Specific to subject or particular aspect of school |
| Perceived Environment Profile [78] | PEP | Does not have recent published psychometrics (>1996) |
| Perceptions of School Social Climate [79] | N/A | Validated with high school sample only |
| I Like School [80] | N/A | Not developed in English |
| Classroom Peer Context Questionnaire [38] | CPCQ | Not a measure of school connectedness  (addressed <5 of 15 components of school connectedness) |
| Classroom Environment Scale [37] | CES | Not a measure of school connectedness  (validated only with high school students, not student self-report) |
| Elementary School Success Profile [81] | N/A | Not specific to school context |
| Scale of Teachers Perception of School Adjustment [82] | PROF-A | Not developed in English |
| California School Climate and Safety Survey [83] | N/A | Not a measure of school connectedness  (addressed <5 of 15 components of school connectedness; did not address behavioural domain) |
| Unnamed (French language questionnaire to measure students perceptions of school context) [84] | N/A | Not developed in English |
| Quality of Life In School [85] | QoLS | Not developed in English |
| Adolescents Sense of Wellbeing Related to Stress [34] | N/A | Not specific to school context |
| Classroom Learning Environment of Elementary Students Questionnaire [86] | CLEES | Not a measure of school connectedness  (not student self-report) |
| Student Support and Student Engagement Scales [87] | N/A | Validated with high school sample only |
| Social Participation Questionnaire [88] | N/A | Specific to children with disabilities |
| Student Engagement in School Scale [89] | N/A | Not a measure of school connectedness  (addressed <5 of 15 components of school connectedness) |
| Student Engagement Scale [90] | N/A | Unable to contact author to request copy of full scale |
| McInerneys Facilitating Conditions Questionnaire [91] | FCQ | Not a measure of school connectedness  (addressed <5 of 15 components of school connectedness) |
| Student Engagement Instrument – Portuguese adaptation [36] | N/A | Validated with high school students only. |
| Classroom Climate Inventory [92] | N/A | Does not have recent published psychometrics (>1996) |
| Quality of School Life [93] | QSL | Unable to contact author to request copy of full scale |
| School Social Climate Questionnaire [94] | CECSCE | Not developed in English |
| Individualized Classroom Environment Questionnaire [95] | ICEQ | Not a measure of school connectedness  (addressed <5 of 15 components of school connectedness) |
| Brief Survey of School Bonding [96] | N/A | Not a measure of school connectedness  (did not address behavioural domain) |
| Unnamed (assesses five aspects of psychosocial classroom environment) [97] | N/A | Not a measure of school connectedness  (not student-self report) |
| School Climate Profile Charles Kettering Ltd. [98] | CFK | Does not have recent published psychometrics (>1996) |
| School Attitude Assessment Survey [99] | SAAS | Validated with high school sample only |
| Students Sense of the School As a Community [100] | N/A | Does not have recent published psychometrics (>1996) |
| Climate4Creativity Student Perspectives Instrument – Elementary and Middle School Version [101] | N/A | Unpublished doctoral dissertation |
| Sense of Belonging to School Scale [102] | SEBES | Unable to contact author to request copy of full scale |
| School Connectedness Survey [103] | N/A | Unpublished doctoral dissertation |
| Constructivist-Oriented Learning Environment Survey [104] | COLES | Validated with high school sample only |
| Unnamed – six items on satisfaction with school [105] | N/A | Not developed in English |
| Unnamed – place identification [106] | N/A | Unable to contact author to request copy of full scale |
| Hemingway Measure of Adolescent Connectedness [107] | N/A | Not specific to school context |
| Questionnaire on Feedback, Identification and School Trajectories [108] | QFITE | Not developed in English |
| Elementary School Ethical Climate Survey [109] | N/A | Not a measure of school connectedness  (not student self report) |
| School Connectedness Scale [110] | N/A | Validated with high school sample only |
| Social-Relational Support for Education Instrument [111] | N/A | Not a measure of school connectedness  (did not address behavioural domain) |
| Unnamed – three scales from Add Health Survey [112] | N/A | Not a measure of school connectedness  (did not address behavioural domain) |
| Georgia Brief School Climate Inventory [113] | GaBSCI | Not a measure of school connectedness  (did not address behavioural domain) |
| School Engagement Measure [114] | N/A | Not a measure of school connectedness  (addressed <5 of 15 components of school connectedness) |
| Invitational School Survey [115] | N/A | Not a measure of school connectedness  (addressed <5 of 15 components of school connectedness) |
| Motivation and Engagement Scale- High School [116] | MES-HS | Not a measure of school connectedness  (addressed <5 of 15 components of school connectedness) |
| Attitudes to School [117] | N/A | Does not have recent published psychometrics (>1996) |
| The Belonging Scale [118] | N/A | Not a measure of school connectedness  (addressed <5 of 15 components of school connectedness; did not address behavioural domain; validated with high school students only) |
| Multidimensional Students Life Satisfaction [119] | N/A | Not specific to school context |
| California School Climate Health and Learning Survey [119] | N/A | Validated with high school students only |
| Quality of School Life [85] | N/A | Does not have recent published psychometrics (>1996) |
| The Saskatchewan School Climate Scale [120] | N/A | Not a measure of school connectedness  (addressed <5 of 15 components of school connectedness) |
| Engagement Versus Disaffection with Learning – Student Report [121] | N/A | Not a measure of school connectedness  (addressed <5 of 15 components of school connectedness) |
| The Behavioural Emotional Cognitive School Engagement Scale [122] | BEC-SES | Not a measure of school connectedness  (addressed <5 of 15 components of school connectedness) |
| Unnamed – school engagement scale [123] | N/A | Not a measure of school connectedness  (addressed <5 of 15 components of school connectedness; validated with high school students only) |
| School Success Profile [124] | SSP | Unable to contact author and request copy of full scale |
| Commitment to School Scale [125] | N/A | Not a measure of school connectedness  (did not address cognitive domain) |
| School Connection Scale [126] | N/A | Not a measure of school connectedness  (did not address behavioural domain; validated with high school students only) |
| School Belonging Scale [127] | N/A | Not a measure of school connectedness  (did not address behavioural domain) |
| Subjective Adjustment Scale [128] | N/A | Not developed in English |
| Socio-Emotional Health Survey [83] | N/A | Not specific to school context |
| Young Children’s Appraisal of Teacher Support [129] | N/A | Not a measure of school connectedness  (addressed <5 of 15 components of school connectedness) |
| Dimensions of Self Concept [130] | N/A | Not a measure of school connectedness  (addressed <5 of 15 components of school connectedness; did not address behavioural domain) |
| Unnamed – student school attitude [131] | N/A | Does not have recent published psychometrics (>1996) |
| Student Attitude Survey [132] | N/A | Does not have recent published psychometrics (>1996) |
| Instructional Climate Survey Form – Student Version [133] | N/A | Not a measure of school connectedness  (did not address behavioural domain) |
| Quality of School Life | N/A | Validated with high school students only. |
| Classroom Life Instrument [134] | CLI | Does not have recent published psychometrics (>1996) |
| Student School Engagement Measure [135] | SSEM | Met eligibility criteria however unable to differentiate between sample that completed Spanish translated version and English version from the data set. |
| School Attitude Questionnaire [136] | SAQ | Not developed in English. |
