# Supplementary material for: Evaluating the psychometric quality of school connectedness measures: A systematic review
Source: PLoS One. 2018 Sep 11;13(9):e0203373. doi: 10.1371/journal.pone.0203373 (PMC6133283; doi:10.1371/journal.pone.0203373)
Supplement: S1 File — (DOCX) [file pone.0203373.s004.docx]

**S1 File. Excluded publications and reasons for exclusion**

An article published by Lam & Jimerson [70] which describes the development of the Student Engagement in Schools Questionnaire (SESQ) was excluded as it was unable to be retrieved.

An article by Hazel, Zavirabadi, Albanes & Gallagher [71] about the 35-item version of the Student Engagement Instrument (SEI) was excluded as authors were unable to differentiate data completed in English and Spanish.

An article by Appleton & Christenson [36] describing the development of the 35-item version of the SEI was excluded as it was an unpublished manuscript.

Authors excluded the Student Bonding Index–Revised manual published by Srivastava and Rodney [72] as it was unable to be retrieved.
